# A Dataset for Electricity Market Studies on Western and Northeastern Power Grids in the United States

**DOI:** 10.1038/s41597-023-02448-w

**Published:** 2023-09-22

**Authors:** Qiwei Zhang, Fangxing Li

**Affiliations:** Dept. of Electrical Engineering and Computer Science, University of Tennessee, Knoxville, USA

**Keywords:** Energy grids and networks, Energy grids and networks

## Abstract

Efficient electricity market operations and cost-effective electricity generations are fundamental to a low-carbon energy future. The Western Electricity Coordinating Council (WECC) and Northeast Power Coordinating Council (NPCC) systems were built to provide efficient electrical grid simulation solutions for their respective U.S. regions. Data reuse for electricity economic studies remains a challenge due to the lack of credible and realistic economic data. This paper delivers a comprehensive dataset containing generator aggregations, generator costs, transmission limits, load distributions, and electricity prices for the WECC and NPCC systems based on real-world grid operation data at year 2020, including power plant geographic locations, generation profiles, regional power flow interchanges, and load distributions in both regions. The electricity price from the developed dataset is simulated based on the other items in the dataset, and we show that the variation of the simulated electricity price reasonably aligns with the real-world electricity price in both the WECC and NPCC regions. Overall, the developed dataset is of interest for various electricity market and economic studies, such as the economic dispatch and locational marginal price (LMP) analysis.

## Background & Summary

Following the U.S. electricity market deregulation started in the 1990s, electric power has been broadly accepted as a tradable commodity, and power grid operations have been coupled with various financial instruments. The physical energy flows are intensively entangled with voluntary monetary exchange, which emphasizes the market-clearing process and the pursuit of future financial returns. Starting with order No. 888, the Federal Energy Regulatory Commission (FERC) has issued more than ten orders to reform and encourage open access to the power grid (https://www.ferc.gov/major-orders-regulations). More than two thirds U.S. electric loads are serviced via electricity markets, where economic signals are applied to guide generation dispatches, congestion managements, and grid investments. The acceleration of the clean energy transition has been rapidly reshaping the generation resource profile. The share of U.S. electricity coming from renewables increased to 29.3% in 2022, and the U.S. aims to achieve net-zero emission by 2050^1^. Considering 25% of total U.S. emission comes from the electricity sector, the clean energy evolution interacts with generation dispatch and market design more powerfully than any other field. Over the past 20 years, technological advances, market deregulation, and the integration of renewable energy have made electricity economics fundamental to reliable energy delivery.

Electricity economics has been widely investigated across various research directions, such as security-constrained economic dispatch^2^, bidding strategy^3^, price analysis^4^, and electricity market cybersecurity^5^. The above research directions rely heavily on grid simulation to demonstrate proposed theories. However, very few datasets have been designed and developed to support demonstrations of electricity economic studies. The IEEE standard system (https://labs.ece.uw.edu/pstca/) library, featuring datasets reduced from real-world electric grids, is the most widely used power grid dataset collection. For example, the IEEE 30 bus system and its dataset represent an approximation of the American Electric Power (AEP) grid as it was in December 1961^6^. However, the IEEE standard test systems are mainly designed for reliability and power flow analysis. A few test systems contain economic data, but they are relatively simple and outdated. The economic data construction in the IEEE standard system library is also non-transparent, which impedes further data reuse and interpretations. To overcome the above electricity economic dataset issues, IEEE formed a working group on test systems for electricity economic analysis in 2007^7^. The working group aims to provide validated datasets for electricity economic studies analogous to the IEEE standard test systems. Under this working group as well as several government-funded projects, a few datasets have been developed to enhance the power system test library with economic data. Li F. and Bo R. have provided an economic dataset for PJM 5-bus and IEEE 30-bus systems focusing on wholesale electricity market price with benchmark results^8^. Billinton R. and Huang D. enhanced IEEE-RTS datasets for adequacy evaluation^9^. Diniz A. L. developed several datasets enhancing the IEEE standard test systems for hydrothermal dispatches^10^. Price J. E. produced a dataset for the Western Electricity Coordinating Council (WECC) system with demand response and energy storage^11^. Palma-Behnke R. developed an electricity economic test system based on the Chilean electric grid^12^. Pena I., Martinez-Anido C. B. and Hodge B. extended the IEEE 118-Bus dataset to accommodate increasing renewables penetration^13^. Texas A&M University provides various synthetic electric grid cases covering most parts of the U.S^14,15^ with comprehensive datasets, including economic data. Although the above datasets enhance power system economic libraries from various perspectives, these datasets more or less suffer from the following three gaps: (1) data construction is non-transparent so it is challenging to make further dataset reuses and modifications; (2) The economic data in these systems may not be constructed using real-world grid operation data, which makes them less suitable for direct comparison or benchmarking against actual electricity market prices; and (3) there is a lack of an up-to-date electricity economic dataset to accommodate the recent generation resource mix.

To bridge the above gaps, we have developed a standardized and realistic dataset for electricity economic studies on the WECC and NPCC systems, including Levelized Cost of Energy (LCOE), generator aggregations, and electricity prices. For the first gap, the construction of this dataset is transparent with raw data available in repository ensuring data reuse. Future users can reuse and modify the raw data to produce a customized and credible dataset based on their research needs. For the second and third gaps, this dataset aggregates generators based on the geographical coordinates of real-world power plants, and they align with the real-world generation resource mix in the year 2020. The generator costs, transmission limits, and load distributions are based on real-world grid operation data from the WECC and NPCC regions, including fuel consumption, fuel price, net generation, regional electricity interflow, and load distributions in the year 2020. The electricity price in this dataset is simulated based on the above data. ***A key feature of this dataset is that the variation of simulated electricity price in the dataset reasonably aligns with the real-world electricity price in the WECC and NPCC regions****.* We provide a benchmark in our repository, which is also described in the *Technical Validation* section below. This consistency between the simulated electricity price and real-world price demonstrates that the dataset provides future users with a flexible and realistic grid simulation platform for demonstrating electricity economic studies, such as economic generation dispatch and electricity market price analysis. The limitation of the dataset can be found at *Limitations* section.

## Methods

### Workflow

**Fig. 1:**
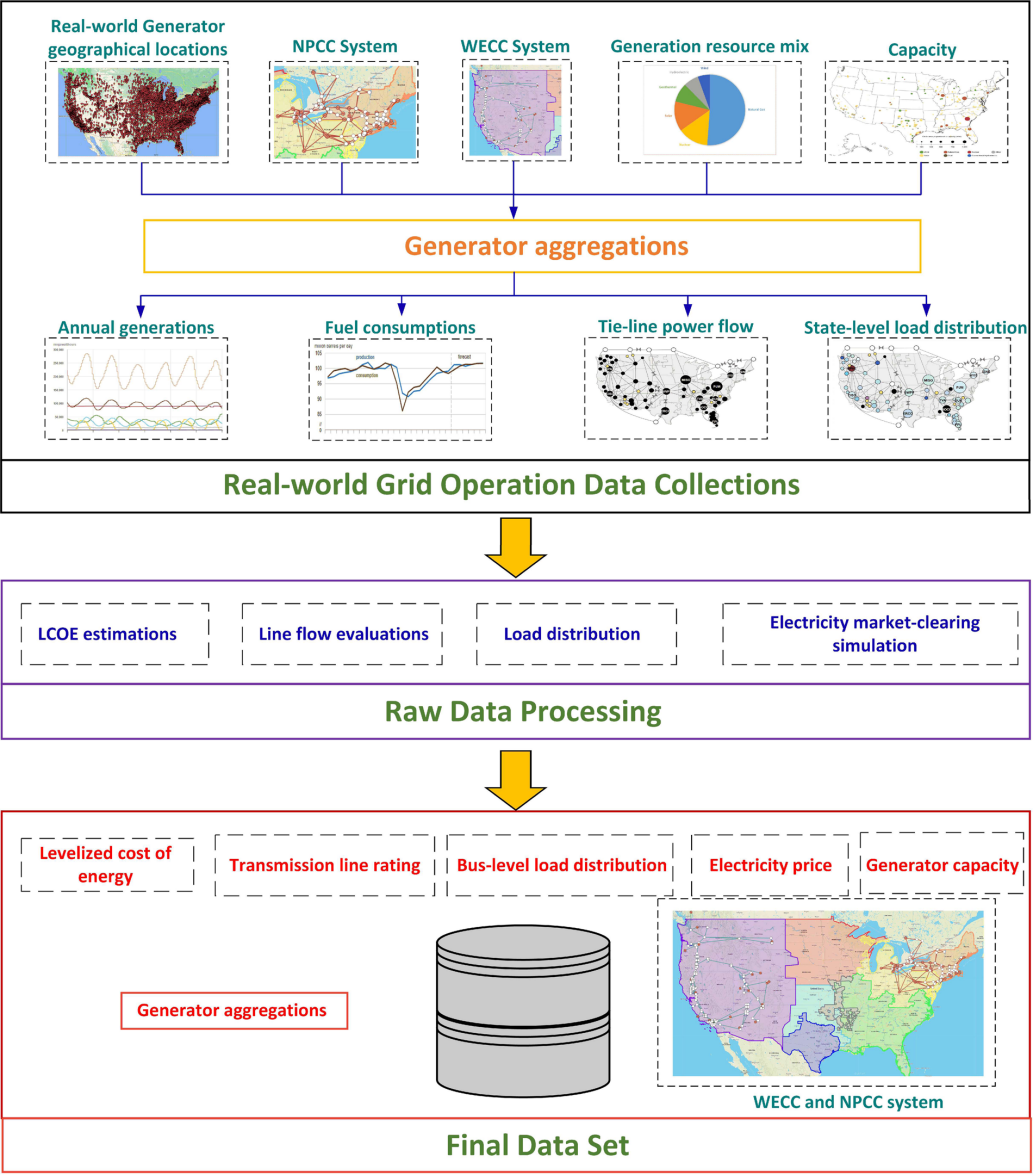
The technical pipeline of the developed dataset. The first block describes the collection of real-world grid operation data and geographic-based generator aggregation. The second block shows the raw data processing to transfer the real-world grid operation data to corresponding data aligning with the WECC and NPCC systems. The last block shows all the items in the dataset. The real-world grid operation data are based on the year 2020. WECC and NPCC systems refer to the test system for WECC and NPCC electric grids. WECC and NPCC regions refers to the real-world WECC and NPCC areas.

In this section, we describe the methods used to create the developed dataset. The technical pipeline for the dataset construction is presented in Fig. 1.

The dataset development can be divided into the following three steps.

- *Step 1 - Generator Aggregation:*
  Power plants in the WECC and NPCC areas are aggregated into different generator buses in the WECC and NPCC systems. We identify and collect the geographical location of all power plants in the WECC and NPCC areas based on Energy Justice Network (http://www.energyjustice.net/map/). The estimated bus locations of the WECC and NPCC systems are provided by the CURENT large-scale testbed (LTB)^16^. Based on the real-world generation resource mix and rule of proximity, different real-world power plants are aggregated to each generator bus in the WECC and NPCC systems.
- *Step 2 - Grid Operation Data Collection:*
  We collect the annual electricity generation, capacity, and fuel consumption for each assigned generator from Energy Information Administration (EIA). The regional power flow interchange and load distribution are collected for different regions within the WECC and NPCC areas. The source of all raw data can be found in Table 7 in Technical Validation Section.
- *Step 3 - Dataset Construction:*
  The generator aggregations and collected real-world grid operation data are used to construct the dataset, including the LCOE, transmission limits, load distribution, and electricity price for the WECC and NPCC systems. LCOE estimation analyzes the fuel price, fuel consumption, and electric generation for selected real-world generators to provide an estimated incremental cost of aggregated generators. The line flow analysis examines real-world power flows to provide an estimation of the transmission line rating. The bus-level load distribution distributes the regional load to buses in the WECC and NPCC systems. With the estimated incremental cost, line rating, capacity, and load distribution, the market-clearing model is simulated to provide electricity prices. The applied real-world grid operation data is based on the year 2020.

### Generator aggregations

**Fig. 2:**
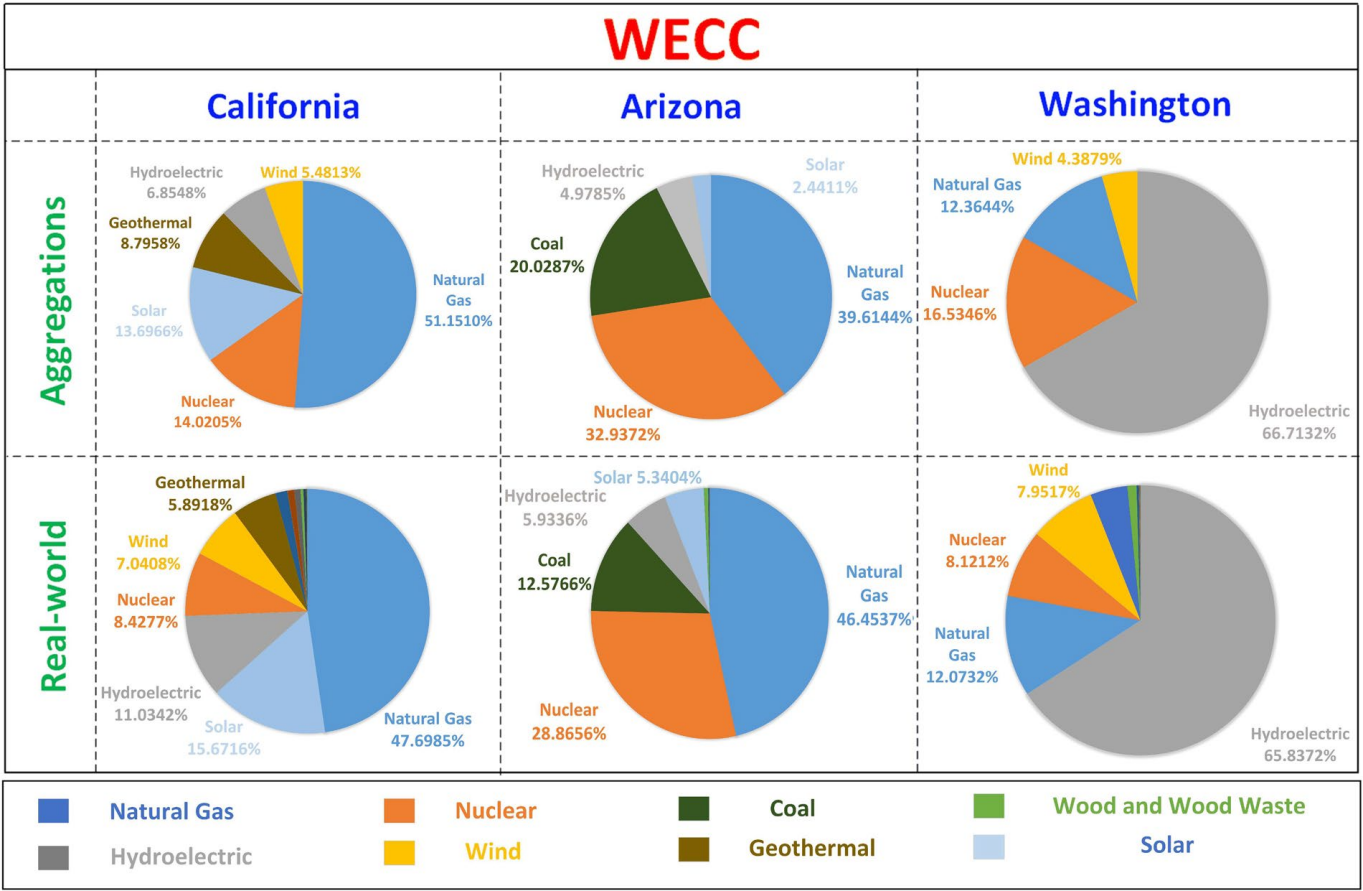
Examples of generation resource mix in the WECC region and aggregated generators in the WECC system.

**Fig. 3:**
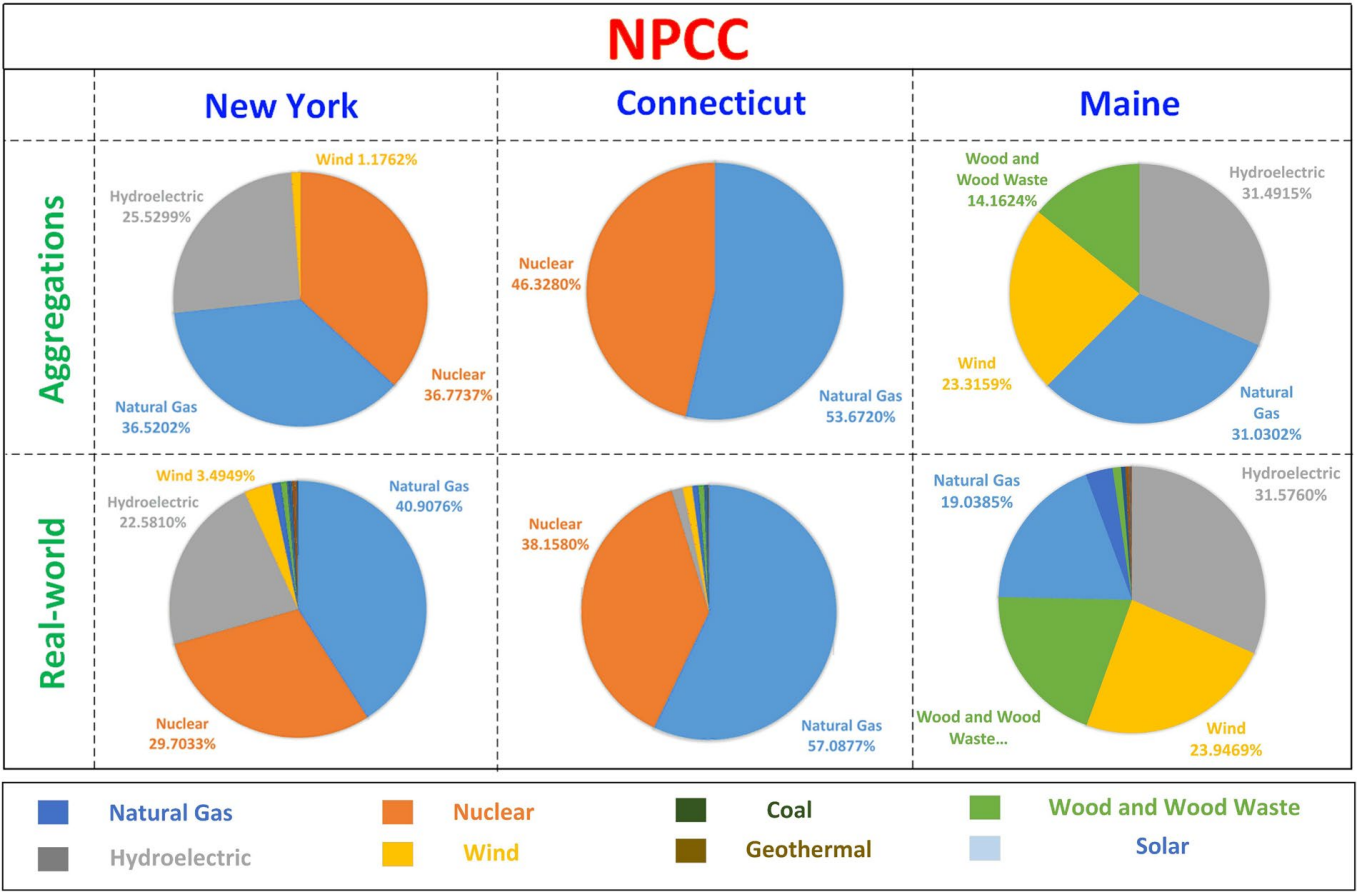
Examples of generation resource mix in the NPCC region and aggregated generators in the NPCC system.

There are over 5000 power plants in the WECC and NPCC regions, and there are 77 generator buses in the WECC and NPCC systems. WECC and NPCC systems refer to the test system for WECC and NPCC electric grids. WECC and NPCC regions refers to the real-world WECC and NPCC areas. To properly assign power plants to each generator bus without loss of generality, generator aggregation is conducted based on generation resource profiles in each state, capacity, and rule of proximity. Figures 2, 3 show that the resource mix of aggregated generators is well aligned with the real-world generators at each state. We list three states in the WECC and NPCC areas as examples in Figs. 2, 3, respectively. The detailed plant names, capacities, and resource mix for the rest of the aggregations can be found in our repository.

It is worth noting that the resource mix of aggregated generators does not match exactly with the resource mix of real-world generators. Power plants with low capacity and generation resources with low percentages are not considered in the aggregation to ensure that future users can easily analyze generator characteristics in their simulations. For example, there is only 0.5% electricity generation from coal power plants in Maine. It is not considered during the generator aggregation because assigning more generators will complicate operation patterns, such as price signals, impeding data reuse. Further, generation aggregation is also based on geographical locations. With the determined resource mix, there are still many selections to aggregate power plants. The WECC and NPCC systems are built based on real-world electric grids in the WECC and NPCC areas^16^, although the details of the system construction remain confidential. Therefore, we search power plants around each bus estimated location in the WECC and NPCC systems and gradually enlarge the search radius until the identified generators match with the generator resource mix at corresponding states. Eventually, each generator bus within the WECC and NPCC systems is configured to include real-world power plants that closely align with the resource mix and geographical coordinates of the region.

### Grid operation data collection

#### Data collection for constructing LCOE and capacity

The dataset construction requires various operational data at WECC and NPCC regions as input. We collect annual net generation and corresponding fuel consumption based on the EIA-923 form. Fuel consumption for different generators is collected with different physical units. For example, gas consumption is measured by *mcf*, diesel consumption is measured in *barrels*, and electricity consumption within the hydro plant is measured by *megawatts*. The annual fuel price for different fuel types is also collected from the EIA. The values of O&M cost are obtained from ISO public data and Statista. The nameplate capacity and nameplate power factor are obtained from EIA-860. The detailed source data can be found in Table 7 in Technical Validation Section.

#### Data collection for constructing load distribution and transmission line rating

**Fig. 4:**
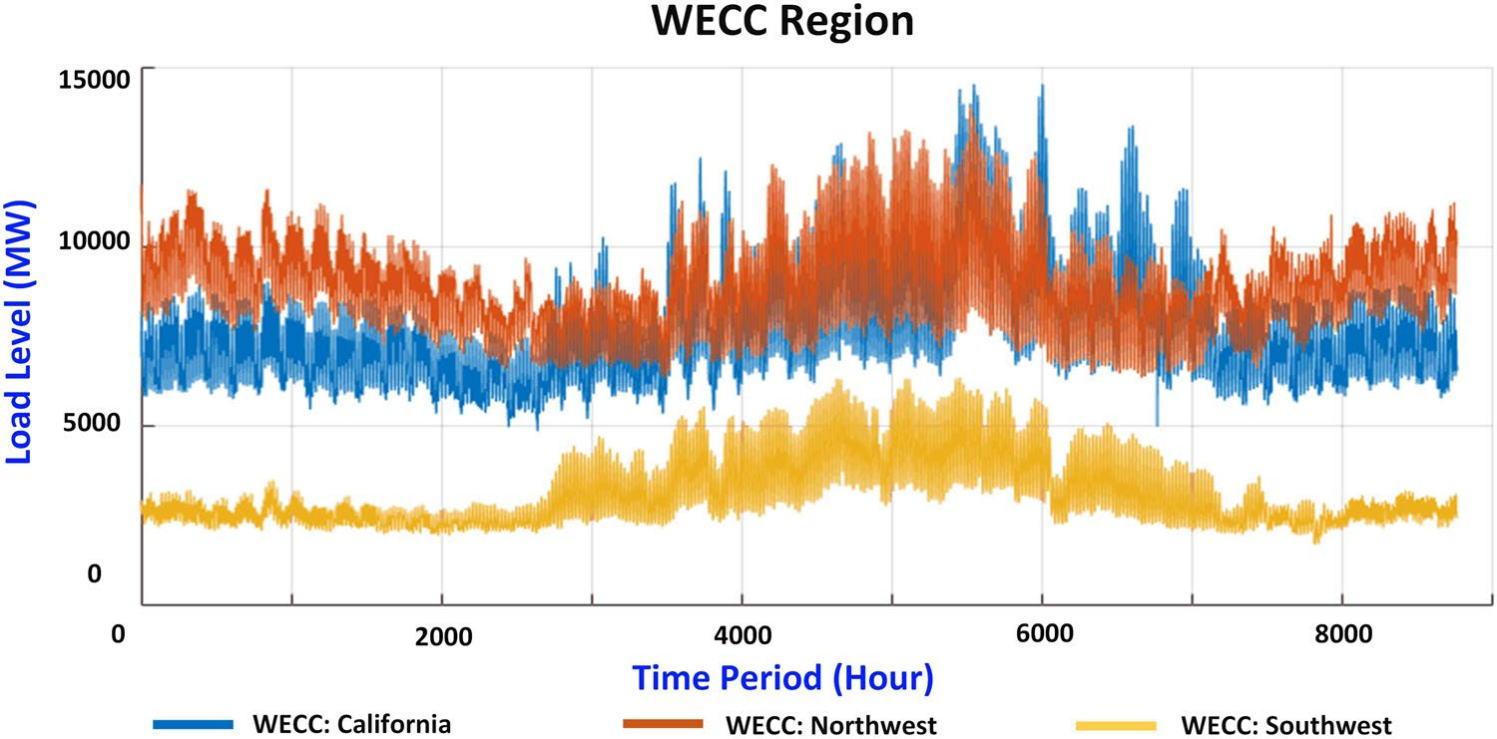
Load distribution for WECC regions.

**Fig. 5:**
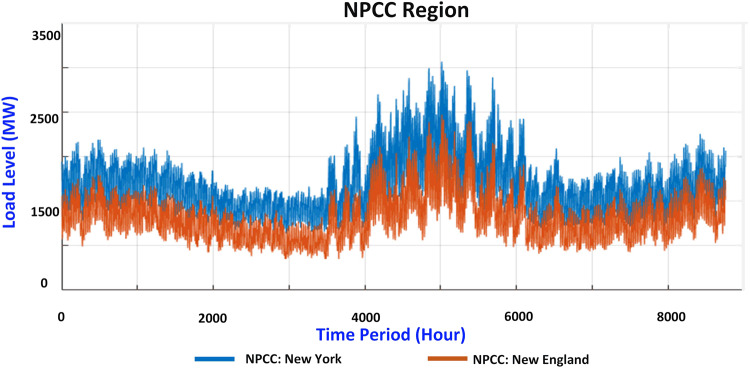
Load distribution for NPCC regions.

The load distribution in this dataset is built based on the real-world WECC and NPCC load distributions in 2020. The WECC region is divided into three areas: California, Northwest Area, and Southwest Area, based on the EIA Electric Grid Monitor (https://www.eia.gov/electricity/gridmonitor/dashboard/electric_overview). Similarly, the NPCC region is divided into two areas: New England and New York. The collected load distribution for the WECC and NPCC areas is shown in Figs. 4, 5, respectively. The electric grid operation data for Canada in the NPCC area is not publicly available, and thus, it is not covered by this dataset. Pennsylvania and Michigan do not belong to the NPCC region, but the NPCC system considers these two areas for completeness.

**Fig. 6:**
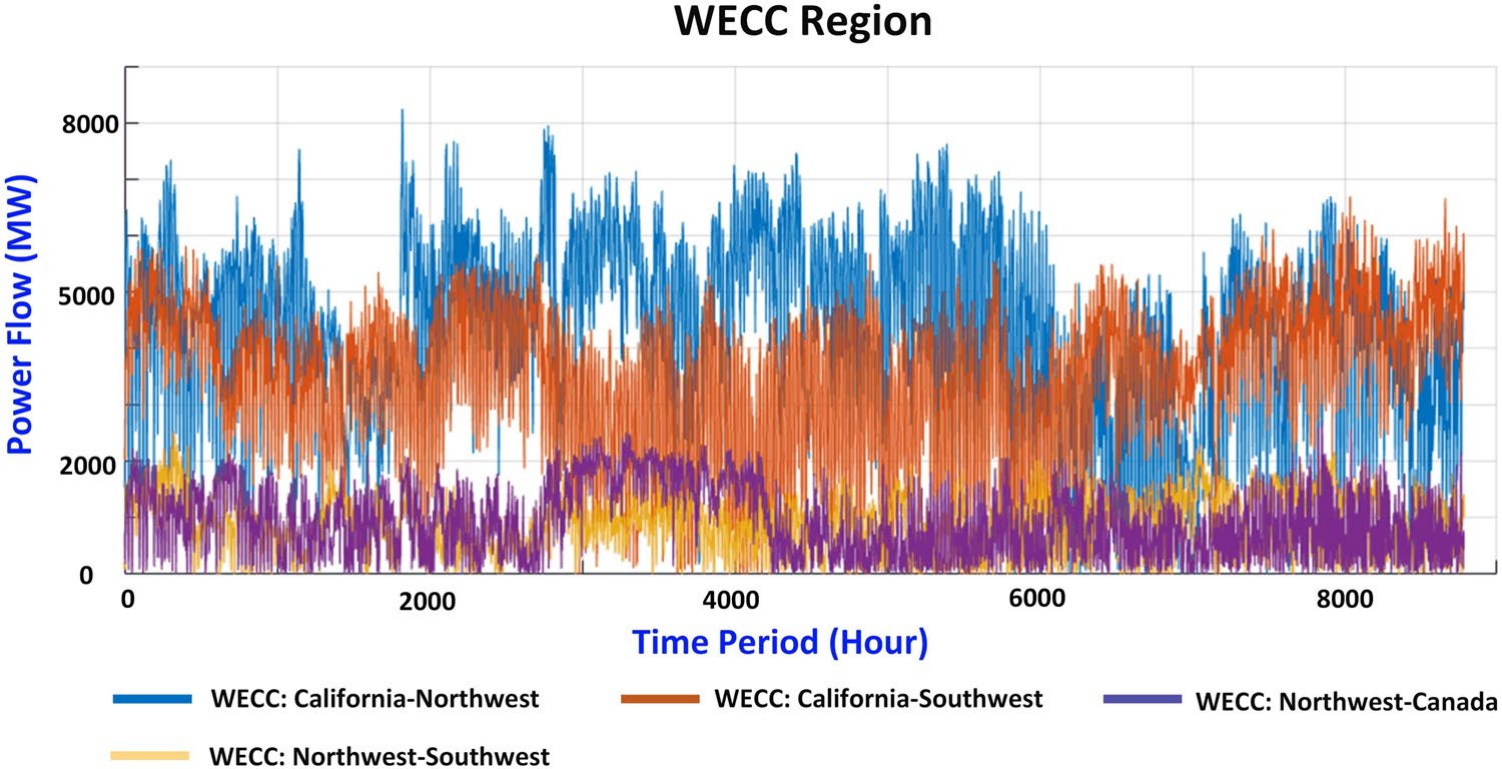
Power flow interchange between different regions in WECC.

**Fig. 7:**
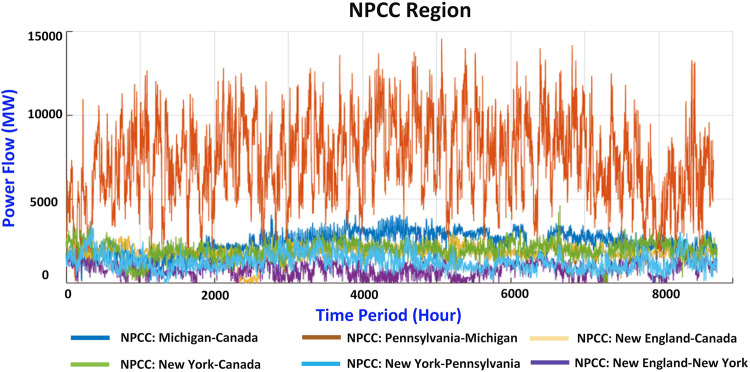
Power flow interchange between different regions in NPCC.

The regional power flow interchanges are also collected from the EIA Electric Grid Monitor. The regional power flow interchange in the WECC and NPCC areas is shown in Figs. 6, 7, respectively. Similar to the regional division in the load distribution part, the WECC region is divided into three areas, and the NPCC region is divided into two areas. The line ratings in Canada, Pennsylvania, and Michigan are included since the power flow interchange data are available.

### Dataset construction

#### Building LCOE and generator capacity

The LCOE is estimated based on the Simple LCOE (sLCOE) calculator developed by the National Renewable Energy Laboratory (https://www.nrel.gov/analysis/tech-lcoe-documentation.html), which allows a comparison of the combination of capital costs, operation and maintenance (O&M) costs, and fuel costs. The LCOE formula is shown in Eq. (1). The first term is depreciation cost, where the *C* represents the overnight capital cost ($), and *Lf* represents the operation life of a power plant measured in years. *P* and *H* represent the net generation output and annual operation hours, respectively. The second term is fuel cost, where *f* represents the fuel consumption, and *f*^*p*^ represents the corresponding fuel cost. For example, *mcf* is used to measure gas consumption in gas-fired units, and the gas price in Jan. 2020 was 3.70 ($/mcf). Multiplying the gas consumption by the gas price gives the cost for purchasing gas. The third term is O&M cost, including fixed O&M cost and variable O&M cost. *OM*_*f*_ represents the annual fixed O&M costs measured in $, which is distributed to each megawatt hour by dividing *P* and *H*. *OM*_*t*_ represents the variable O&M costs measured in $/MWh, which is the incremental maintenance cost.

1 $${\rm{COE}}=\mathop{\underbrace{\frac{C}{Lf\cdot P\cdot H}}}\limits_{Depreciation}+\mathop{\underbrace{\frac{f\cdot {f}^{p}}{P\cdot H}}}\limits_{fuel}+\mathop{\underbrace{\frac{O{M}_{f}}{P\cdot H}+O{M}_{t}}}\limits_{O\& M}$$

LCOE in Eq. (1) is designed for a single unit. Given the generator aggregation, the Eq. (1) can be reformulated into Eq. (2), which is applied to obtain the LCOE for each aggregated generator.

2 $${\rm{COE}}=\mathop{\underbrace{\frac{\sum _{i\in A}C}{\sum _{i\in A}Lf\cdot {P}_{i}\cdot H}}}\limits_{Depreciation}+\mathop{\underbrace{\frac{\sum _{i\in A}\,f\cdot {f}^{p}}{\sum _{i\in A}{P}_{i}\cdot H}}}\limits_{fuel}+\mathop{\underbrace{\frac{\sum _{i\in A}O\mathop{M}\limits_{f}}{\sum _{i\in A}{P}_{i}\cdot H}+\frac{\sum _{i\in A}O{M}_{t}\cdot {P}_{i}}{\sum _{i\in A}{P}_{i}}}}\limits_{O\& M}$$

According to the generator aggregation, the generator capacity at each generator bus is obtained by Eq. (3), where *Cap*_*i*_^*n*^ and *f*_*i*_^*n*^ represent nameplate capacity and nameplate power factor, respectively.

3 $${{\rm{Cap}}}_{g}=\sum _{i\in A}Ca{p}_{i}^{n}{f}_{i}^{n}$$

The collection of all parameters in (1), (2), and (3) is described in the *Gird Operation Data Collection*.

#### Building load distribution and transmission line rating

The load distribution is estimated by the collected regional load at WECC and NPCC regions, the estimated locations of system load buses, and load participation factor of system load buses. The collected system load buses are firstly grouped by regions based on geographic locations. Then, the collected real-world loads are distributed to load buses located within the corresponding region based on the given load participation factor. The bus-level load is obtained by multiplying the regional level load with corresponding load participation factors.

The line rating of inter-area transmission lines in the WECC and NPCC systems are estimated by corresponding average power flow interchanges. The real-world power flow interchanges are not directly applicable as the line rating since the simulation system in the dataset simplifies the real-world generations, and if we use it directly, it is very likely that there is no congestion during almost all time periods. Power system economic studies sometimes prefer congestion to test new models under different congestion scenarios. Therefore, we use the following procedure to result in congestion in the simulation during peak load scenarios. The average power flow interchanges are equally divided and assigned to each inter-area transmission line as the initial line rating. Weight factors are assigned to the initial line rating at each inter-area line, and we gradually decrease weight factors from 1 until the 10% inter-area lines are congested when the loading level is at 80% of its maximum. Future users may modify the factor to customize the congestion status.

#### Building electricity price

U.S. wholesale electricity markets are organized by independent system operators (ISOs) and usually consist of day-ahead and real-time markets. For both day-ahead and real-time market operations, security-constrained unit commitment, security-constrained economic dispatch (SCED), and LMP schemes are employed to determine the generation dispatches and market settlements. The electricity price in this dataset is simulated by the SCED and LMP model. A standard SCED and LMP model used by ISOs is shown in Eqs. (4–8)^17^. Objective (4) minimizes the generation dispatch cost. Constraint (5) ensures the electric generation-load balance. Constraints (6) and (7) ensure generations and line flows are within corresponding limits. Variables *C*_*i*_*, D*_*i*_*, P*^*max*^*, P*^*min*^*, L*_*l*_^*max*^, and *L*_*l*_^*min*^ represent the cost, load, generation capacities, and transmission line limits, respectively. *GSF*_*l-i*_ represents the generation shift factor, which indicates grid topology information. *N*_*G*_ and *N*_*b*_ represent the number of generators and buses. The LMP is calculated along with the SCED result, as shown in Eq. (8). The U.S. electricity market applies LMP to determine the wholesale electricity price at different locations. The *λ* represents the Lagrangian multiplier of power balance constraint (5). The *τ*_*i*_^+^ and *τ*_*i*_^*−*^ represent Lagrangian multipliers of the positive and negative transmission line limits (7).

4 $$\min \mathop{\sum }\limits_{i}^{{N}_{G}}{C}_{i}{P}_{i}$$

5 $$\mathop{\sum }\limits_{i=1}^{{N}_{b}}{P}_{i}-\mathop{\sum }\limits_{i=1}^{{N}_{b}}{D}_{i}=0\,:\lambda $$

6 $${P}_{i}^{\min }\le {P}_{i}\le {P}_{i}^{\max },\forall i\in {N}_{b}\,:{\eta }^{-},{\eta }^{+}$$

7 $${L}_{l}^{\min }\le \mathop{\sum }\limits_{i=1}^{{N}_{b}}GS{F}_{l-i}({P}_{i}-{D}_{i})\le {L}_{l}^{\max }\forall l\in {N}_{L}\,:{\tau }^{+},{\tau }^{-}$$

8 $$LM{P}_{i}=\lambda -\mathop{\sum }\limits_{l=1}^{{N}_{l}}GS{F}_{l-i}({\tau }_{i}^{+}-{\tau }_{i}^{-})\quad \forall i\in {N}_{b}$$

The previous subsections have built the cost, load, generation capacities, and transmission line limits for the WECC and NPCC systems, which are inputs to the model (4)-(7). Then, LMPs can be calculated for the WECC and NPCC systems based on Eq. (8).

## Data Records

The developed dataset and detailed instructions are available in figshare^18^ and GitHub repositories. In Github, two root folders are created: the **Source Data** folder contains all the original source files used to generate the developed dataset; and the **WECC and NPCC Systems** folder stores the complete developed dataset described in this paper. In figshare, subfolders are not supported as of the time when this work is completed, therefore, two RAR files are uploaded in figshare corresponding to the **Source Data** and **WECC and NPCC Systems** folders, respectively. The following discussion is based on the Github structure or the files unzipped from the two RAR files in figshare.

A description of the **Source Data** is also provided in the repository. In the **WECC and NPCC Systems** folder, subfolders are organized by data type, and are described as follows. Tables 1–6 are examples for each indicator that helps to clarify further the types of data and how the indicator looks like.

**Table 1:** Example of aggregated generator information in *NPCC_A.xlsx* and *WECC_A.xlsx*.

Clay (50)			
Plant Name	Net Energy (MWh)	Total Fuel Consumption (MMBtu)	Fuel Type
Nine Mile Point Nuclear 1	10,167,608	106,210,612	NUC
Nine Mile Point Nuclear 2	5,473,000	57,170,841	NUC
*Aggregate*	15,640,608	163,381,453

**Table 2:** Elements in *Capacity.xlsx*.

Generator ID	Capacity (MW)

**Table 3:** Elements in *Line_rating.xlsx*.

Line ID	From Bus	To Bus	Limits (MW)

**Table 4:** Elements in *Load.xlsx*.

Time	1	2	…
Bus ID			
1	Load at bus 1 time 1	Load at bus 1 time 1	Load at bus 1 time …
…	Load at bus … time 1	Load at bus … time 2	Load at bus … time …

**Table 5:** Elements in *Cost.xlsx*.

Generator ID	Incremental cost	Fixed cost

**Table 6:** Elements in *LMP.xlsx*.

Time	1	2	…
Bus ID			
1	Price at bus 1 time 1	Price at bus 1 time 1	Price at bus 1 time …
…	Price at bus … time 1	Price at bus … time 2	Price at bus … time …

**Folder**: Generator Aggregation

**File**: *NPCC_ Aggregation.xlsx* and *WECC_ Aggregation.xlsx*

These files stores generator aggregation information for the NPCC/WECC systems. The files provide a list of detailed real-world power plants that are aggregated to each bus in the NPCC/WECC systems. Pie chart comparisons between the real-world generation resource mix and aggregated resource mix are also provided at each state within NPCC/WECC areas. Specifically, the excel sheet include many sheets, which are named by the state name (e.g., New York) and the state name with suffix “Aggregate” (e.g., New York Aggregate). The sheet with the state name describes the generation resource mix for that state before the aggregation. The sheet with suffix “Aggregate” describes the aggregated generator information. An example of aggregated generator information is shown in Table 1. The first row “Clay (50)” is the name of system bus with bus number 50. The “Plant Name” indicate the real-world power plants that are aggregated into this generator bus. The “Fuel Type” indicate the generator type of power plants. “Net Energy” and “Total Fuel Consumption” are the yearly generations and fuel consumptions.

**Folder**: Capacity

**File**: *Capacity.xlsx*

This file stores the capacity in megawatt (MW) of aggregated generators in sheet ‘NPCC’ and sheet ‘WECC’ for the NPCC and WECC systems, respectively. As shown in Table 2, the first column of the excel sheet shows the generator ID indicating the order of the generator in the system, and the second column of the excel sheet shows the capacity of the aggregated generator in MW.

**Folder**: Line Rating

**File**: *Line_Rating.xlsx*

This file records the line ratings (in MW) in sheets ‘NPCC’ and ‘WECC’ for the NPCC and WECC systems, respectively. The line ID, from bus, and end bus are also provided in each column, respectively, as shown in Table 3.

**Folder**: Load

**File**: *Load.xlsx*

This file provides a year-round hourly load in MW for each bus in the NPCC and WECC systems. The data is stored in the sheets ‘NPCC’ and ‘WECC’, respectively. As shown in Table 4, the first column of the sheet shows the bus ID, and the second row of the sheet shows the time in terms of hours. The value at a cell is the load amount at corresponding bus ID and time.

**Folder**: Cost

**File**: *Cost.xlsx*

This file stores the cost (dollar, $) of aggregated generators under LCOE in the sheets ‘NPCC_LCOE’ and ‘WECC_LCOE ‘ for the NPCC and WECC systems, respectively. As shown in Table 5, the first column of the sheet shows the generator ID. The second column of the sheet is the incremental cost, and the third column of the sheet is the fixed cost. Similarly, this file stores the cost (dollar, $) of aggregated generators under operational cost in the sheets ‘NPCC_Cost’ and ‘WECC_ Cost’ for the NPCC and WECC systems, respectively.

**Folder**: Price

**File**: *LMP.xlsx*

This file provides a year-round hourly electricity price (dollar) for each bus in the NPCC and WECC systems. The data is stored in the sheets ‘NPCC’ and ‘WECC’, respectively. The first column shows the bus ID, and the second line shows the corresponding hour.

**Folder**: Network Topology

**File**: *Network_Topology_NPCC.xlsx* and *Network_Topology_WECC.xlsx*

These two files provide topology of bus-branch connectivity for NPCC and WECC systems, respectively.

**Folder**: System Matpower Format

**File**: *NPCC.m* and *WECC.m*

MATPOWER case files^6^ are built based on the developed datasets for the NPCC/WECC systems for user’s convenience. User can access the datasets by loading the ‘NPCC.m’ or ‘WECC.m’ case file with MATPOWER function ‘loadcase()’

**File**: *NPCC_load.m* and *WECC_load.m*

A ‘.mat’ type load file is also provided for users’ convenience on applying the load with MATPOWER on MATLAB.

**File**: *NPCC_LMP.m* and *WECC_LMP.m*

A ‘.mat’ type load file is also provided for users’ convenience on investigating the price on MATLAB.

## Technical Validation

The developed dataset contains generator aggregation, LCOE, transmission line rating, load distribution, generator capacity, and simulated electricity price for the WECC and NPCC systems. The quality of source data is validated by the EIA and other well-known organizations, as shown in Table 7. It is also worth noting that the dataset could be customized based on raw data from other sources other than the sources listed in Table 7, as long as the raw data are qualified (or preferred by future users).

**Table 7:** Source data of the developed dataset.

Item in the dataset	Real-world raw data	Source file
Generator aggregation	System topology and bus coordinates	Large-scale Testbed^16^ (upon request) and Homeland Infrastructure Foundation Level Database(https://hifldgeoplatform. opendata.arcgis.com/datasets/transmission-lines)
	Power plant coordinates	Energy Justice Network (http://www.energyjustice.net/map)
	Renewable energy resources	EIA-923 Form (https://www.eia.gov/electricity/data/eia923)
Generator capacity	Generator capacity	EIA-860 Form (https://www.eia.gov/electricity/data/eia860/)
LCOE	Fuel consumption	EIA-923 Form
	Annual generation	
	Fuel price	EIA Price Data (https://www.eia.gov/petroleum/data.php#prices) and (https://www.eia.gov/electricity/state)
	O&M cost	U.S. O&M cost (https://www.statista.com/statistics/519144/power-plant-operation-and-maintenance-costs-in-the-us-by-technology/) and (http://www.caiso.com/documents/variableoperationsandmaintenancecostreport-dec212018.pdf)
Transmission line rating	Inter-region power flow	EIA BA-to-BA interchange (https://www.eia.gov/electricity/gridmonitor/dashboard/electric_overview)
Load distribution	Load distribution	EIA Demand (https://www.eia.gov/electricity/gridmonitor/dashboard/electric_overview)
Electricity price	N/A (Simulated)	Standard market-clearing model^16^

It describes the collected real-world grid operation data and corresponding source files.

In addition to the source data, we provide a validation test to demonstrate the quality of the developed dataset. The electricity price in this dataset is simulated based on the other items in the dataset, as described in the *Method* section. Therefore, to demonstrate the quality of the developed dataset, we compared the simulated electricity price in the dataset with the real-world electricity price in 2020 in the same geographical location.

**Fig. 8:**
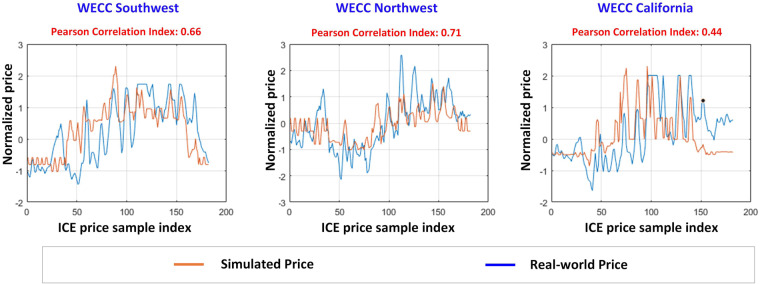
Comparisons between the simulated price and real-world price for WECC. The left most subfigure shows the simulated price and real-world price at WECC Southwest area. The middle subfigure shows simulated price and real-world price at WECC Northwest area. The right most subfigure shows simulated price and real-world price at WECC California area.

**Fig. 9:**
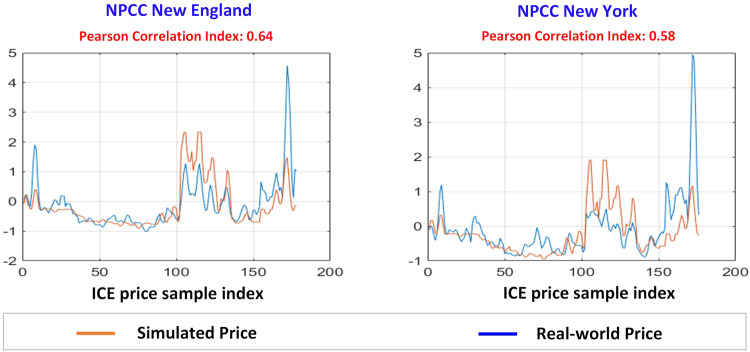
Comparisons between the simulated price and real-world price for NPCC. The left subfigure shows simulated price and real-world price at NPCC New England. The right subfigure shows simulated price and real-world price at NPCC New York.

Through Intercontinental Exchange (ICE), the EIA provides the daily wholesale electricity price at four hubs located in the WECC southwest, WECC northwest, and WECC California areas spanning the year 2020 (https://www.eia.gov/electricity/wholesale/). The simulated price data at buses located in the above three regions are averaged to be compared with the ICE price as shown in Fig. 8. Both prices are normalized by a z-score with center 0 and standard deviation 1 to reduce the impact of price magnitude. The simulated price and ICE real-world price have similar price shapes, as shown in the orange and blue curves, respectively. The comparison is also performed on the NPCC system, as shown in Fig. 9. The EIA does not provide wholesale electricity prices for New York state (https://www.eia.gov/electricity/wholesale/). Therefore, the wholesale electricity price in New York state is obtained from NYISO (https://www.nyiso.com/energy-market-operational-data) with the same timestamp as ICE. The variation of the simulated price is relatively consistent with the variation of the real-world price for the NPCC area.

Based on the results, the Pearson values for the correlation between the simulated price and ICE real-world price are calculated for all WECC and NPCC areas (i.e., WECC southwest, WECC northwest, WECC California, NPCC New England, and NPCC New York), which are 0.66, 0.71, 0.44, 0.64, and 0.58, respectively. The average is 0.60 which is interpreted as “moderate positive” correlation between the simulated and real-world prices^19^. It is worth noting that the correlation value for California (0.44) is slightly lower than the correlation value for other areas. The real-world electricity price is impacted by various factors that cannot be reflected by the simulated price, such as contingency events or unplanned outages. Therefore, the simulated price may have lower consistency with the real-world price in some regions. However, the overall pattern in the simulated price and the average Pearson correlation value demonstrate that the developed dataset is reasonably aligned with the real-world electricity prices in the WECC and NPCC regions.

## Limitations

### Limitation 1

The presented dataset is built based on real-world operation data for the year 2020, and the provided load distribution and corresponding simulated price data are in hourly resolution. Therefore, the dataset can accurately reflect the operational condition of the year 2020. It is worth noting that the provided dataset aims to provide researchers with a simulation dataset for power system economic studies to demonstrate their theories and algorithms, instead of providing a real-world dataset for real-world analysis. One of the key characteristics of this dataset is a transparent data construction process, such that users can customize the dataset based on their needs. For example, future users can replace the raw data in 2020 from this dataset with new raw data from 2025 to customize the datasets that are more closely relevant to the operating condition of year 2025.

### Limitation 2

The incremental LCOE in this dataset did not capture some of the factors such as inflations and capital recovery factors. The LCOE in this dataset only considers a generalized depreciation cost, O&M cost, and fuel cost. The detailed cost decomposition, such as nuclear waste disposal cost, is not specifically represented in the dataset. Also, the LCOE could be higher than the operation cost since it includes capital cost. Future users can customize the LCOE formula to integrate or remove a particular cost that they are interested in their specific economic study.

### Limitation 3

The line rating in the dataset is not directly based on the real-world inter-area power flow but is redesigned to create congestion during peak load scenarios (see *Dataset Construction*). Future users may modify this process to customize the congestion status based on their specific research needs.

### Limitation 4

The dataset aims to provide simulation test cases for power system economic studies, where future users can demonstrate their new theories and models. Future research that applies the developed dataset will have a simulated price reasonably aligned with the real-world price variation. However, it is worth noting that there are many factors that cannot be captured by this dataset, such as contingency events or unplanned outages of generators and transmission. Also, the real-world marginal generation pattern is much more complicated than the marginal generation pattern presented in our dataset since the real-world generators are aggregated in the dataset. Thus, the goal of this dataset is to provide a relatively realistic simulation test case for power system economic studies, instead of developing a digital twin of real-world power grids. Future developers could use this dataset as a cornerstone for customization and modification to obtain their own test systems that may closely map real-world operating conditions for specific scenarios.

### Limitation 5

The generator aggregation relies on the CURENT LTB testbed for estimated bus locations. The CURENT LTB testbed provides well-constructed topology data, and this testbed received the R&D 100 award in 2020 (https://www.rdworldonline.com/rd-100-2020-winner/curent-ltb/) and various publications have demonstrated the quality of the testbed, such as literature^16,20^.

## Usage Notes

The collected source data and the developed dataset presented in this paper can be downloaded from our repository. Users can follow the detailed instructions on repository for data reuse. The geographic coordinates for each bus in the WECC and NPCC systems have restricted access, but users can contact the CURENT research center to request the information.

## Data Availability

Relevant code and detailed instructions associated with this manuscript can be found in the figshare repository (10.6084/m9.figshare.24116484) and GitHub repository (https://github.com/enliten/ENLITEN-Grid-Econ-Data).
